# Acute Respiratory Failure in Allergic Bronchopulmonary Aspergillosis Characterized by Diffuse Lung Lesions: A Case Report and Literature Review

**DOI:** 10.1111/crj.70115

**Published:** 2025-07-22

**Authors:** Hongmei Ren

**Affiliations:** ^1^ Department of Respiratory and Critical Care Medicine, Yangpu Hospital, School of Medicine Tongji University Shanghai China

**Keywords:** acute respiratory failure, allergic, bronchopulmonary aspergillosis, diffuse lung lesions

## Abstract

Allergic bronchopulmonary aspergillosis (ABPA) is an allergic lung disease caused by sensitivity to *Aspergillus fumigatus*. Diffuse lung lesions as a radiological presentation of ABPA are exceedingly rare, with no documented case in the literature. We present a case with asthma. High‐resolution computed tomography (HRCT) of the chest revealed diffuse lung lesions. Additionally, arterial blood gas analysis revealed life‐threatening acute Type II respiratory failure. Initially, there was suspicion of a mycobacterial infection. However, a subsequent diagnosis revealed the atypical presentation of ABPA. Finally, the patient's symptoms improved, and lung shadows were absorbed after undergoing mechanical ventilation with tracheal intubation and receiving methylprednisolone treatment.

## Graphical abstract

In this work, we present a case of allergic bronchopulmonary aspergillosis. High‐resolution computed tomography (HRCT) of the chest revealed diffuse lung lesions. Additionally, arterial blood gas analysis indicated life‐threatening acute Type II respiratory failure.

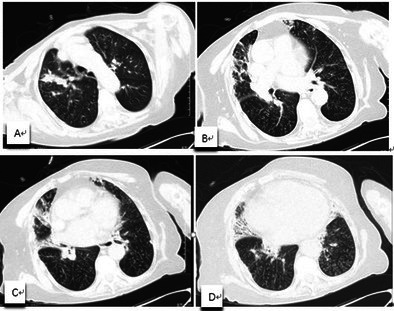

## 1 Introduction

Allergic bronchopulmonary aspergillosis (ABPA) is a hypersensitivity disorder triggered by antigens from the *Aspergillus* species, primarily *Aspergillus fumigatus*. It involves immune reactions against the fungus [1], constituting a complex allergic condition. The common radiological abnormalities include fleeting pulmonary opacities, bronchiectasis, and mucoid impaction. Patients in Stage V may experience progressive inflammation and fibrous cavity lesions caused by airway dilation, which can lead to evolving respiratory failure and death [2]. We report the first case of ABPA presenting as acute Type II respiratory failure secondary to diffuse lung lesions.

## 2 Case Report

A mid‐80s‐year‐old woman presented to our hospital with a week‐long history of coughing, sputum production, and fever. The patient has been diagnosed with bronchial asthma for over 20 years and has been receiving long‐term treatment with salmeterol/fluticasone (50/250 μg) 100/500 μg per day via inhalation, effectively managing the condition. The patient's medical history includes previous occurrences of coronary heart disease, diabetes, and hypertension. The patient lives on the first floor, and her living environment is humid. Upon examination, the patient's blood pressure was measured at 155/71 mmHg. Various wheezing sounds and coarse rales were audible in both lungs.

**FIGURE 1:**
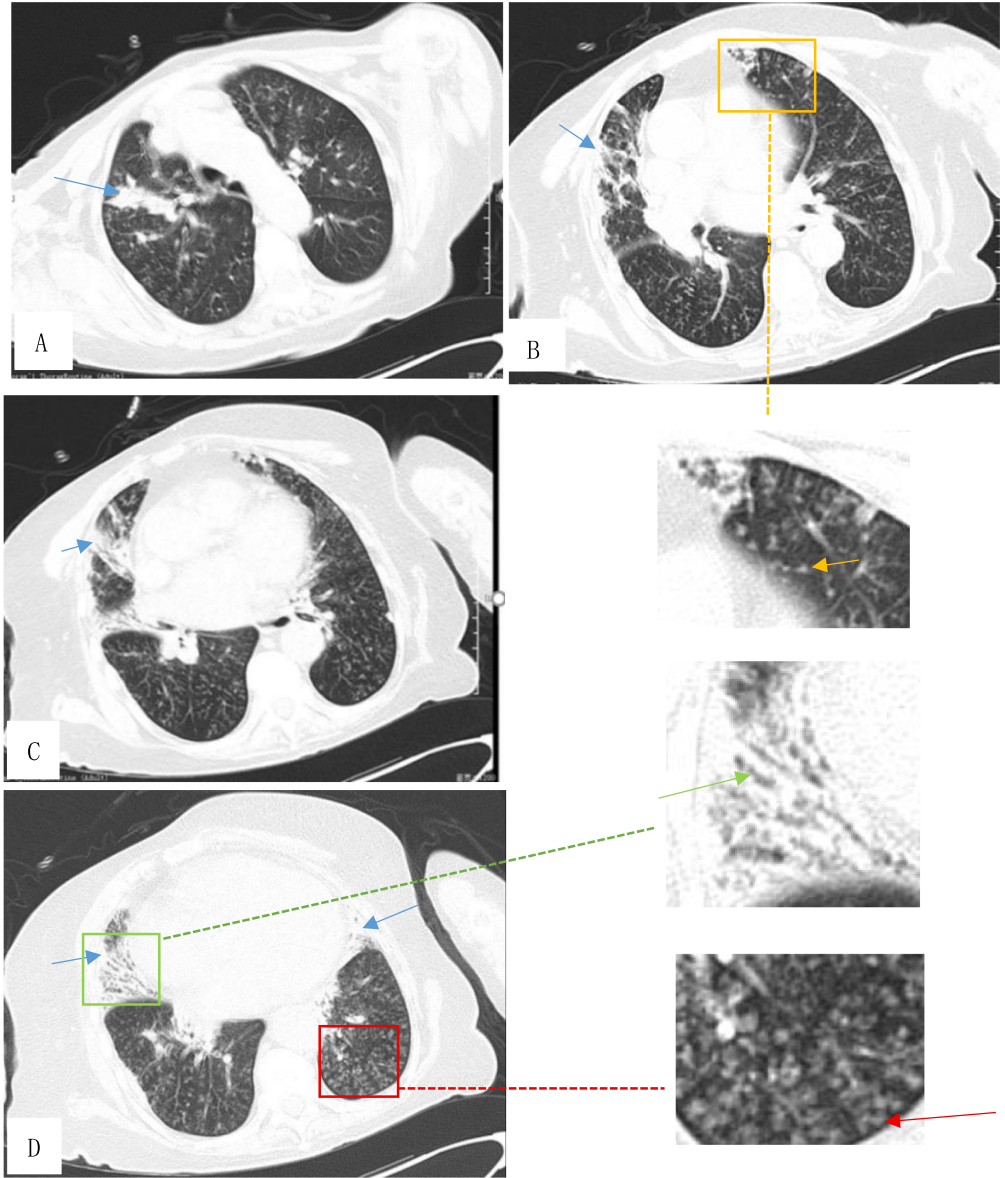
(A–D) D1 HRCT of the chest showed diffuse lung lesions: diffuse miliary nodules opacities (orange arrow) and “tree‐in‐bud” patterns (red arrow) distributed throughout both lung fields, infiltration shadows (blue arrow) in both upper lungs and the middle lobe of the right lung, as well as traction bronchiectasis (green arrow) in the right middle lobe.

HRCT of the chest (Figure 1A–D) revealed diffuse lung lesions. Blood routine examination demonstrated 13.5 × 10^9^/L of white blood cells (WBC), 112 g/L of hemoglobin (Hb), and 0.36 × 10^9^/L of eosinophils (EOS). Arterial blood gas showed a pH of 7.155, a PCO_2_ of 16.10 kPa, and a PO_2_ of 19.4 kPa. Her urine and fecal routine, liver and kidney function, and myocardial enzymes were normal. Her B‐type natriuretic peptide (BNP) was measured at 194 pg/mL (reference value < 100 pg/mL).

The patient underwent emergency tracheal intubation and received mechanical ventilation treatment. Empirical antibiotic therapy was initiated with Imipenem 1 g every 12 h and nalidixic acid 500 mg once a day.

Subsequently, her glycosylated hemoglobin was normal. Results for rheumatic immune indicators were negative. The cold agglutination test, acid‐fast bacilli in sputum, T‐SPOT.TB, IgM antibody determination for nine pathogens, and throat swab nucleic acid test for six pathogens all yielded negative results. Total serum immunoglobulin E (IgE) level is elevated at 17 900.0 IU/mL (reference value < 100 IU/mL), and IgE specific for *A. fumigatus* is elevated at 1.97 kUA/L (reference value < 0.35 IU/mL).

Finally, the possibility of Mycobacterium infection, diffuse panbronchiolitis, and heart failure was excluded. The diagnosis of ABPA was confirmed, as outlined in the Expert Consensus on the Diagnosis and Treatment of Allergic Bronchopulmonary Aspergillosis in 2022 [3]. The patient was initiated on a methylprednisolone injection at a dose of 40 mg. Eight days later, PCO_2_ significantly decreased to 6.95 kPa. The tracheal intubation was removed, and noninvasive ventilation with a BiPAP mask was provided.

**FIGURE 2:**
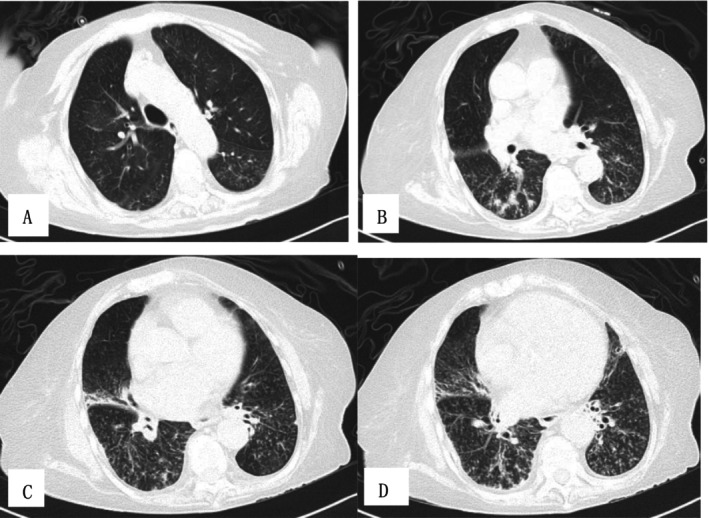
(A–D) D20 chest HRCT showed significant absorption of lesions in both upper lobes, while miliary nodules opacities and “tree‐in‐bud” patterns in the lower lobes progressed.

Two weeks after admission, PaCO_2_ decreased to 5.21 kPa. Blood EOS were 0 × 10^9^/L. Total IgE decreased to 13 100 IU/mL, and specific IgE for *A. fumigatus* decreased to 1.38 kUA/L. The dosage of methylprednisolone was reduced to 20 mg once daily. After 20 days of treatment, upon reexamination of the chest CT, significant absorption of lesions was observed in both upper lobes, while the presence of miliary nodules opacities and “tree‐in‐bud” patterns in the lower lobes had increased (Figure 2A–D). The patient was discharged from the hospital 1 month after admission. The dosage of methylprednisolone was reduced to 12 mg once daily.

**FIGURE 3:**
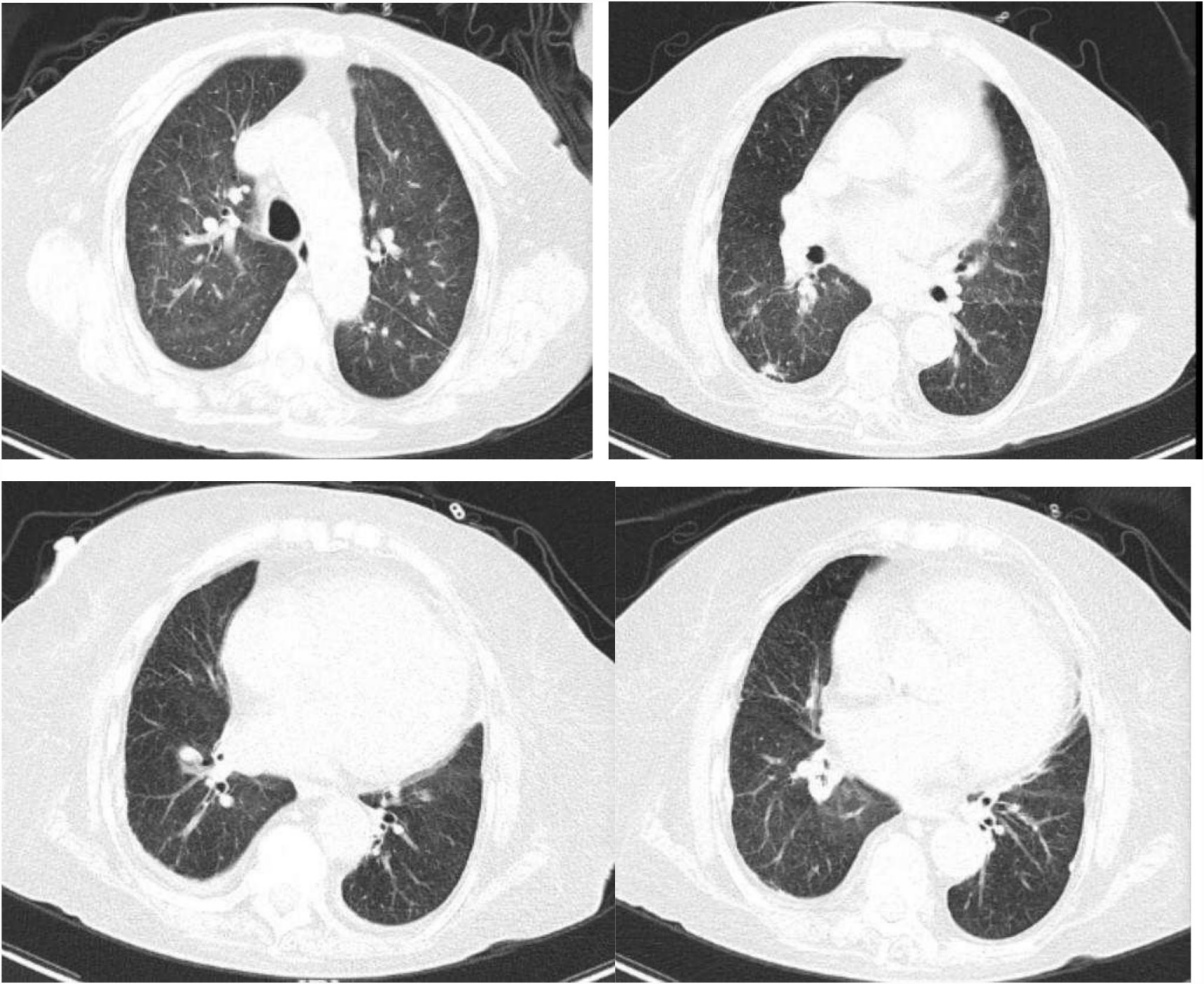
(A–D) After 3 months, a chest HRCT showed significant absorption of lesions in both lungs.

After 3 months of treatment, she only mentioned experiencing mild dyspnea during physical exercise. HRCT of the chest revealed significant absorption of lung lesions (Figure 3A–D). Total IgE dropped to 1335 IU/mL, specific IgE of *A. fumigatus* decreased to 0.44 kUA/L, and blood EOS increased to 0.83 × 10^9^/L. Arterial blood gas analysis showed normal results (Table 1). The dosage of methylprednisolone was reduced to 8 mg once per day.

**TABLE 1:** Data of this case.

Age (year)	Mid‐80s‐year‐old			
Gender (male/female)	Female			
History	Asthma			
Date	D1	D14	D20	After 3 months of treatment
Chest radiograph	Diffuse lung lesions	NA	Significant absorption of lesions was observed in both upper lobes, while the presence of miliary nodules opacities and “tree‐in‐bud” patterns in the lower lobes had increased	Significant absorption of lung lesions
Total IgE (IU/mL)	17 900	13 100	NA	1335
Serum S IgE (kUA/L)	1.97	1.38	NA	0.44
Peripheral blood eosinophils (10^9^/L)	0.36	0	NA	0.83
Arterial blood PH	7.155	7.403	NA	7.430
Arterial blood PCO_2_ (kPa)	16.10	5.21	NA	5.17
Arterial blood PO_2_ (kPa)	19.40	14.5	NA	12.4
Treatment	Methylprednisolone 40 mg per day	Methylprednisolone 12 mg per day	Methylprednisolone 12 mg per day	Methylprednisolone 8 mg once per day
Prognosis	Good			

## 3 Discussion

ABPA is an allergic lung disease caused by *A. fumigatus* allergy. It is characterized by bronchial asthma and recurrent lung shadows, which may result in bronchiectasis. ABPA is often misdiagnosed or overlooked in clinical practice. The disease remains underdiagnosed in many countries, and as many as one‐third of cases are misdiagnosed as pulmonary tuberculosis [4]. Early diagnosis and timely appropriate treatment can help manage the condition and prevent irreversible bronchopulmonary damage [1, 5]. Late‐stage ABPA patients exhibit lesions such as irreversible bronchiectasis, pulmonary fibrosis, and pulmonary cavities, as observed on chest imaging. These conditions can result in respiratory failure and, in severe cases, may lead to death [6].

*Aspergillus* conidia, due to their small diameter (2–3 μm), can easily reach the pulmonary alveoli and deposit there. *A. fumigatus* is the most common ubiquitous airborne fungus and the causative organism for ABPA [7]. Exposure to environments with high concentrations of fungal spores, such as damp buildings, can easily result in ABPA [8]. Individuals at risk of developing ABPA are primarily patients with asthma or cystic fibrosis. Rarely, ABPA occurs in individuals with chronic obstructive pulmonary disease [9]. The thick mucus in the airways of these patients makes it difficult to clear the *Aspergillus* spores when inhaled.

The diagnosis of ABPA relies on the association of suggestive symptoms, biological markers, and radiologic findings. HRCT scan is the effective imaging modality for diagnosing ABPA, as it can detect bronchiectasis, mucoid impaction, centrilobular nodules, tree‐in‐bud opacities, mosaic attenuation, and pleuropulmonary fibrosis [10]. Uncommon radiological manifestations of ABPA include miliary nodules [8, 11, 12], large pulmonary masses [13], and pleural effusions [14].

Among the four patients with ABPA combined with acute respiratory failure that we reviewed [15, 16, 17, 18], chest imaging reveals patchy consolidation and atelectasis. The uniqueness of this case lies in two aspects. On one hand, HRCT revealed diffuse lung lesions characterized by widespread miliary nodular opacities and “tree‐in‐bud” patterns distributed throughout both lung fields. Additionally, infiltration shadows were observed in both upper lungs and the middle lobe of the right lung, along with traction bronchiectasis in the right middle lobe. On the other hand, this case was accompanied by life‐threatening acute respiratory failure.

Oral glucocorticoids are the cornerstone of ABPA treatment. Three cases were treated with glucocorticoid therapy. Antifungal drugs exert a therapeutic effect by reducing fungal colonization in the airways and decreasing the inflammatory response. Three out of the four cases were treated with antifungal medication. Due to life‐threatening respiratory failure, two cases were treated with mechanical ventilation via endotracheal intubation, and one of them received V‐V ECMO treatment. Our patient underwent mechanical ventilation via endotracheal intubation and received methylprednisolone therapy. Mechanical ventilation via endotracheal intubation is a critical intervention in the management of respiratory failure.

The prognosis for all cases was positive. After 3 months of treatment, the patients' symptoms improved, the chest shadows were significantly absorbed, and serum IgE levels decreased significantly.

In conclusion, when encountering a case of bronchial asthma presenting with diffuse lesions in both lungs alongside respiratory failure, consideration should be given to the possibility of ABPA. Early diagnosis and management of ABPA will help prevent the development of end‐stage pulmonary fibrosis.

## Author Contributions

H.R. contributed to the conception, design, data collection, analysis, interpretation of data, and writing of the article.

## Ethics Statement

Our study was approved by the ethics committee of Yangpu Hospital, School of Medicine, Tongji University.

## Conflicts of Interest

The author declares no conflicts of interest.

## Data Availability

The data that support the findings of this study are available from the corresponding author upon reasonable request.
